# Syndrome de Scott et grossesse: à propos d’un cas

**DOI:** 10.11604/pamj.2023.44.151.38361

**Published:** 2023-03-29

**Authors:** Allae Eddine Bouchaib, Mamadou Alpha Balde, Abdellah Babahabib, Moulay El Mehdi Elhassani, Jaouad Kouach

**Affiliations:** 1 Service de Gynécologie-Obstétrique, Hôpital Militaire d´Instruction Mohamed V, Rabat, Maroc

**Keywords:** Thrombopathies, Scott, grossesse, hémorragie, cas clinique, Thrombopathies, Scott, pregnancy, hemorrhage, case report

## Abstract

Le syndrome de Scott est une thrombopathie congénitale rare, de transmission autosomique récessive. Notre travail a pour objectif de rapporter le cas d´une parturiente porteuse d´un syndrome de Scott secondaire à une mutation du gène ANO6 jamais décrite dans la littérature. Après 3 épisodes hémorragiques surtout après un avortement spontané, Le diagnostic a été confirmé par une cytométrie en flux. Cette patiente a été suivie tout au long de sa grossesse dans notre formation puis a accouché par voie basse à terme sous mesures préventives particulières avec des suites simples pour la mère et le nouveau-né. Le traitement du syndrome de Scott est purement symptomatique et fait appel principalement aux transfusions de plaquettes. Puisque c´est une thrombopathie rare à haut risque hémorragique, avec un diagnostic difficile, la grossesse et l´accouchement chez les patientes porteuses de ce syndrome nécessitent une surveillance rigoureuse vu que le pronostic vital est engagé.

## Abstract (en)

Scott´s syndrome is a rare congenital thrombopathy with an autosomal recessive inheritance pattern. The purpose of our study is to report the case of a parturient woman with Scott´s syndrome secondary to a mutation of the ANO6 gene never described in the literature. After 3 bleeding episodes, in particular after spontaneous abortion, the diagnosis was confirmed by flow cytometry. This patient was monitored during pregnancy in our centre and then gave birth via vaginal delivery at full term. Special preventive measures were taken. Outcome was good for the mother and the newborn. The treatment of Scott´s syndrome is purely symptomatic and mainly involves platelet transfusions. Since it is a rare thrombopathy with a high bleeding risk and difficult diagnosis, pregnancy and childbirth in patients with this syndrome require rigorous monitoring, as the prognosis is life threatening.

## Introduction

Le syndrome de Scott (SS) est une thrombopathie congénitale rare de transmission autosomique récessive; dû à un défaut d´expression sur la membrane des plaquettes activées, de la phosphatidylsérine (PS) [1]. Il se traduit cliniquement par un syndrome hémorragique cutanéomuqueux provoqué, d´intensité variable. Biologiquement, la normalité du bilan d´hémostase de première intention complique la démarche diagnostique. Le SS est évoqué devant un effondrement du test de consommation de la prothrombine et le diagnostic est confirmé par la cytométrie en flux qui met en évidence un défaut d´expression de la PS après activation plaquettaire [2]. Nous rapportons un cas d´une patiente ayant un syndrome de Scott et porteuse d´une mutation du gène ANO6 jamais décrite dans la littérature diagnostiquée 5 ans avant sa grossesse, retrouvée à l´état homozygote au niveau de l´exon 12 du gène ANO6. La grossesse a été suivie au service de gynécologie-obstétrique de l´Hôpital Militaire d´Instruction Mohamed V de Rabat, suivie d´un accouchement par voie basse, sous mesures préventives particulières sans aucun incident hémorragique.

## Patient et observation

**Figure 1:**
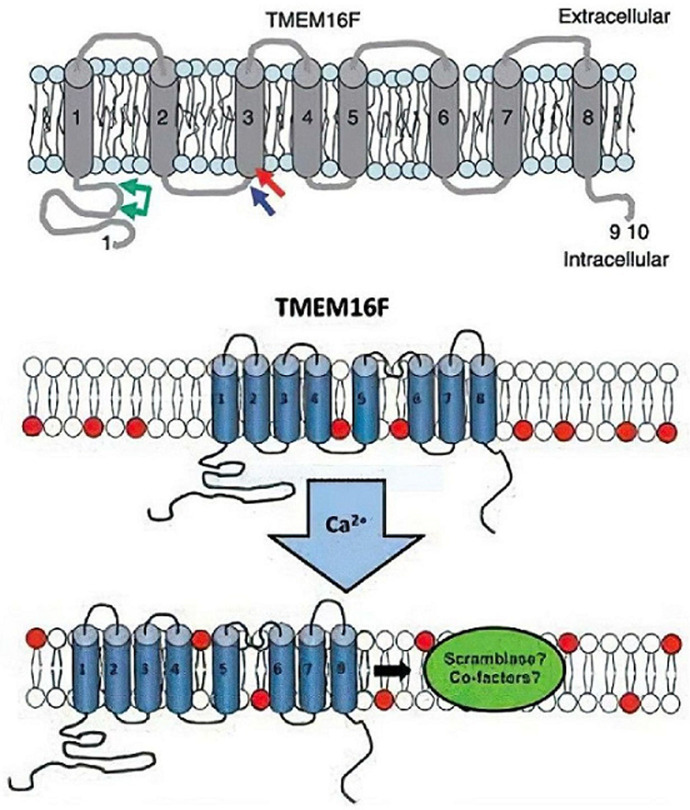
activation de la phospholipide scramblase

**Informations relatives de la patiente**: il s´agit d´une patiente âgée de 36 ans, groupage A Rh+, G2P0, rapportant une notion de consanguinité de 1^er^ degré chez les parents, suivie pour thrombopathie de Scott depuis 5 ans, ayant bénéficié d´une extraction dentaire qui s´est compliquée d´un hématome du palais il y´a 3 ans. La patiente a présenté un épisode de métrorragie sans retentissement clinique, avec un antécédent d´avortement spontané précoce à 2 mois non aspiré ni cureté compliquée d´une métrorragie prolongée; pour cela un bilan a été demandé objectivant un mécanisme défectueux de la scramblase, qui expose la PS pro coagulante sur la surface externe des plaquettes sanguines activées (Figure 1).

**Chronologie**: cinq ans après la confirmation du diagnostic du syndrome de Scott, la patiente s´est présentée pour accouchement; avec une deuxième grossesse estimée à 41 SA selon DDR précise et échographie de 12 SA, suivie dans notre formation ayant bénéficié de plusieurs CPN et échographies sans particularités avec un bilan prénatal complet et correct; à noter qu´au cours de cette grossesse la patiente n´a pas présenté de syndrome hémorragique.

**Figure 2:**
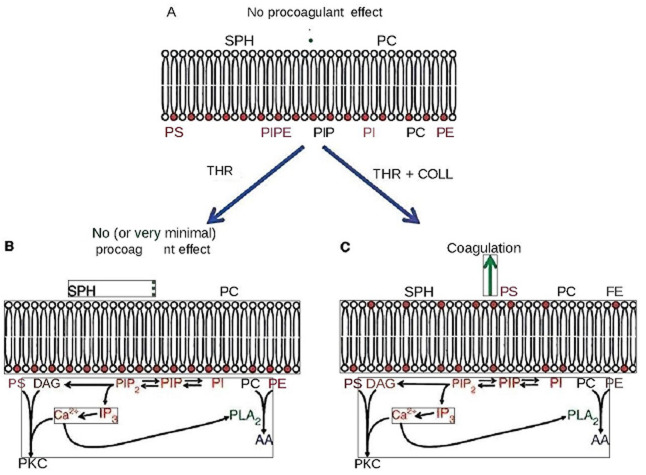
vue schématique de la distribution transversale des phospholipides dans la membrane plasmique des plaquettes au repos et stimulées

**Figure 3:**
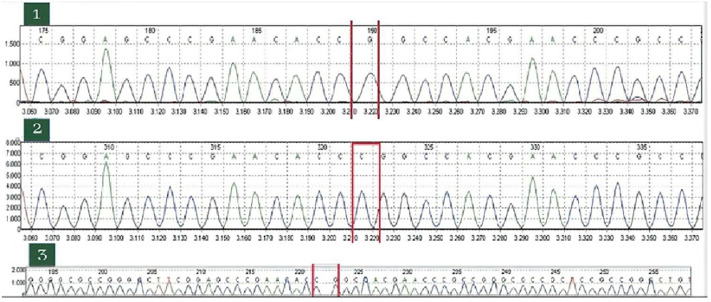
chromatogrammes de séquence illustrant la mutation dans la protéine anoctamine 6

**Figure 4:**
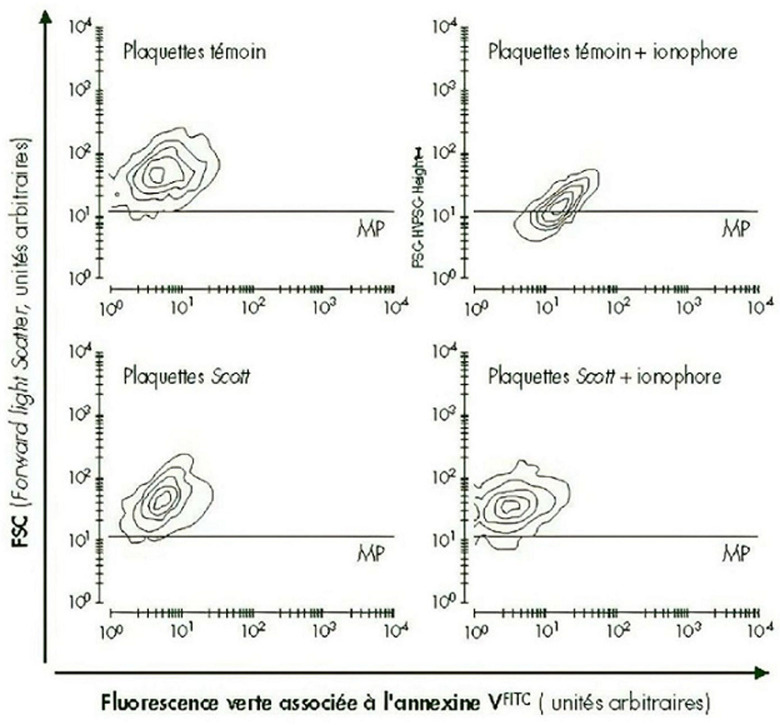
cytométrie en flux confirmant l'absence d'expression de plaquettes Scott sur la surface plaquettaire activée

**Démarche diagnostique**: devant le risque hémorragique important, un suivi en hémato a été réalisé avec un bilan de 1^e^ et 2^e^ intention marqué par un TCA allongé avec rapport TCA patient sur TCA témoin de 1.34; un discret déficit en facteur XII (à 39%); un taux et aspect des plaquettes avec test d´agrégation: normaux et un test de consommation de la prothrombine diminuée (Figure 2). Le diagnostic du syndrome de Scott a été reconfirmé après réalisation d´un séquençage illustrant la mutation dans ANO6 (Figure 3) et d´une cytométrie en flux (Figure 4); l´analyse des données cliniques et para cliniques de la patiente a été faite en coordination entre services de gynécologie obstétrique et hématologie pour préparer l´accouchement. Elle consulte à 41 SA en phase de latence du travail.

**Résultats cliniques**: l´examen clinique et obstétrical lors de l´admission de la patiente a objectivé une hauteur utérine à 32 cm, des bruits cardiaques fœtaux positifs et réguliers avec 2 contractions par 10 minutes, par ailleurs l´examen au speculum ne trouve pas de saignement, ni d´hydrorrhées.

**Intervention thérapeutique**: la péridurale étant contre-indiquée, une analgésie par l´association du néfopam et du paracétamol a été reçue par voie intra veineuse, au cours de la surveillance le constat cervical est passé de 80% 2d à 4 cm au bout de 45mn puis à dilatation complète en 1h, suivi d´un accouchement par voie basse en occipito-pubienne sans épisiotomie ; d´un nouveau-né de sexe M, PDN 3620g, APGAR 10/10, délivrance artificielle sous sédation devant la rétention placentaire. Durant l´accouchement, la prise d´anti fibrinolytiques était systématique (acide tranexamique 1 g toutes les 8 heures en IV puis relai per os) pendant le travail et 48 heures après l´accouchement. A titre préventif, la patiente a bénéficié de la transfusion de 2 culots plaquettaires avant l´accouchement et 2 culots plaquettaires après l´accouchement puis 2 culots globulaires.

**Suivi et résultats des interventions thérapeutiques**: les suites post partum ont été simples pour la mère et le nouveau-né sans aucun incident hémorragique. Elle a été mise sous contraception à base de microprogestatif à partir du 21^e^ jour du post partum.

**Consentement éclairé**: la patiente est consentante pour la publication de son cas clinique après avoir été avisé de l´intérêt scientifique de partager sa pathologie rare.

## Discussion

Le syndrome de Scott est une thrombopathie héréditaire rare de transmission autosomique récessive due à un défaut d´expression de la phosphatidylsérine sur la membrane des plaquettes activées [3]. Les cas décrits dans la littérature montrent une prédominance du sexe féminin; sa prévalence est estimée à 1/1 000 000 [4]. A ce jour, seuls 6 cas atteints du syndrome de Scott ont été publiés dans la littérature, dont 4 ont bénéficié d´un séquençage du gène ANO6 [5], et c´est le cas de notre patiente. Les mécanismes physiopathologiques du SS ne sont pas bien élucidés [6]. Sur le plan cellulaire Le syndrome de Scott est un trouble héréditaire de la coagulation associé au maintien de l'asymétrie de la bicouche lipidique dans les membranes des cellules sanguines, y compris les plaquettes [7]. L'exposition de la PS par les plaquettes activées est particulièrement induite par l'activation de la scramblase, une enzyme capable de générer une redistribution bidirectionnelle [5]. Génétiquement, le diagnostic de SS chez notre patiente a été mis en évidence à travers un séquençage haut débit utilisant le séquenceur MiSeq (Illumina), la mutation c.1318C>T (p. (Arg440*) à l´état homozygote dans l´exon 12 du gène codant la protéine anoctamine-6 (ANO6) aboutissant à un codage type non-sens pour la première fois. Il s´agit d´une mutation du gène ANO6 jamais décrite dans la littérature. Notre parturiente a des parents consanguins.

Généralement le SS résulte de mutations dans ANO6, qui code pour la protéine phospholipide scramblase (TMEM16F) qui est située sur le bras long du chromosome 12 (12q12) [8].

Comme c´est le cas chez notre patiente, avec une bonne tolérance clinique, ce syndrome est probablement sous diagnostiqué vu la présentation clinique relativement bénigne et les difficultés diagnostiques ainsi que la normalité des tests de première intention explorant l'hémostase primaire [4]. Les sujets hétérozygotes à la mutation d´ANO6 sont asymptomatiques. Notre patiente a présenté un épisode de gingivorragies avec avortement hémorragique, et c´est le cas pour les rares patientes décrites dans la littérature qui ont présenté des saignements de nez, des saignements de gencives, des hématuries, des pétéchies, des saignements menstruels abondants et des hémarthroses [9]. En cas de suspicion clinique de syndrome de Scott, il faut d'abord éliminer les autres troubles de la coagulation ainsi que les autres thrombopathies tels que le pseudo Willebrand, et la maladie de Bernard soulier [10]. Notre patiente a bénéficié d´une cytométrie en flux qui permet de détecter l´absence ou le déficit d´exposition de la PS à la surface des plaquettes activées, confirmant ainsi le diagnostic.

La grossesse chez les femmes atteintes de syndrome de Scott est une occurrence peu fréquente qui pose un défi en termes de gestion des risques hémorragiques durant la grossesse, l'accouchement et le post-partum immédiat, qui sont des moments critiques. Jusqu'à présent, aucune étude ne recommande de déconseiller la grossesse chez les femmes souffrant de thrombopathies congénitales rares. Les patientes porteuses d´un syndrome de Scott décrites dans la littérature jusqu'à présent sont toutes des femmes et les 5 patientes ayant eu des enfants [4] ont toutes souffert d'une hémorragie du postpartum, ayant nécessité une hystérectomie d´hémostase avec deux décès maternels, alors chez notre patiente l´accouchement s´est déroulé sans complication hémorragique lors d´un accouchement par voie basse mené à terme sans épisiotomie, sachant qu´elle a eu un syndrome hémorragique après le 1^er^ avortement.

Le traitement du syndrome de Scott n´est pas codifié, il doit être supervisé par une équipe pluridisciplinaire dédiée. Les transfusions plaquettaires ont été utilisées avec succès dans les cas publiés dans la littérature ainsi que chez notre patiente. L´utilisation de concentrés plaquettaires déleucocytés permet la prévention de l´alloimmunisation anti-HLA et donc la survenue d´un état réfractaire aux transfusions plaquettaires itératives [3,5]. Le conseil génétique serait un moyen intéressant de dépistage des thrombopathies constitutionnelles, surtout dans les situations d´histoire familiale de syndrome hémorragique.

## Conclusion

Le syndrome de Scott est une maladie rare, dont les mécanismes physiopathologiques commencent à peine à être élucidés. La grossesse et l´accouchement chez la patiente porteuse d´un syndrome de Scott nécessite des mesures préventives et curatives particulières vu le risque hémorragique élevé surtout en post partum. Notre patiente porteuse du syndrome de Scott a pu mener une grossesse à terme suivie d´un accouchement par voie basse sans incidents hémorragiques. La gestion de la grossesse chez les femmes atteintes de SS est toujours délicate, d’où la nécessité d´une prise en charge au niveau d´un centre pluridisciplinaire capable de faire le diagnostic de cette maladie et de gérer complications maternelles et fœtales ou cours de la grossesse.
